# Buffalo Medical and Surgical Association

**Published:** 1890-01

**Authors:** W. H. Bergtold

**Affiliations:** Secretary


### BUFFALO MEDICAL AND SURGICAL ASSOCIATION.

Reported by W. H. BERGTOLD, M. D., Secretary.

REGULAR meeting, held Tuesday, December 3d, at 8.30 P. M., in the Library of the Polytechnic Institute, No. 9 West Mohawk street.

The President, Dr. A. A. HUBBELL, in the chair.

Dr. CHARLES E. CONGDON was proposed for membership.

Dr. HERMAN MYNTER presented a case of arthrectomy of the knee.

The patient, a boy about ten years old, was first seen on June 15, 1889. He gave a history of having been healthy until about April 15th, when he was suddenly taken with severe pains in the right knee, fever, etc. He had been in bed up to June 15th, was emaciated, had an evening rise of temperature; there was atrophy of the thigh and calf, constant pain, and much lateral motion. The knee was a typical case of so-called white swelling. At the operation it was admitted by those present that the case was hopeless, and that it was useless to try to save the leg. Through the usual transverse incision, Dr. Mynter was able to determine that the trouble had probably started as a central necrosis of the epiphysis; that the whole external condyle was destroyed, and that there was a large bone abscess, the lower end of the femur containing a sequestrum. As much of the joint capsule as possible was removed, together with the patella, and all involved bone was scraped out. The leg was dressed to get Schede's aseptic blood-clot, and immobilized by a plaster-of-Paris bandage dressing.

After seven weeks, during which time there had been no rise of temperature, the primary dressing was removed. Perfect union by first intention was found. The boy has since continued to improve, feels well, and walks with ease. There is a tendency, however, to some *genu valgum*, and Dr. Mynter suggested that this might be due to the growth of the remaining internal epiphyseal cartilage.

DISCUSSION.

Dr. Hayd thought Dr. Mynter's explanation of the *genu valgum* condition was not a good one, for he did not believe that sufficient growth could occur in six months to produce this condition. It was, in his opinion, caused by the removal of the external condyle.

Dr. Howell thought that the idea of splintering the leg, in its present condition, was a good one. This would keep the leg straight until the soft growing structures had time to become firm by calcification.

Dr. Tremaine believed that there is always, normally, a tendency to overgrowth of the internal condyle, and that this fact, together with the removal of the external condyle, was ample and sufficient explanation of the present condition. If necessary, a MacEwen's operation could be done later, to correct the deformity. The present case was a highly successful one, and reflected much credit on the operator.

Dr. M. B. Folwell, having been called out of town, was unable to present his paper.

Dr. S. Y. Howell then read on The Etiology and Relation of Chorea to Other Diseases, and The Pathology of Chorea; and was followed by Dr. H. D. Ingraham, who read on The Symptoms, Prognosis, and Treatment of Chorea.

DISCUSSION.

Dr. J. W. Putnam—Chorea is liable to be undiagnosticated, and, on the other hand, often stated to be present when not. We frequently see children who are brought for treatment, with the idea that they have chorea. They go through a few motions, as raising the shoulders, the corners of the mouth, or moving the scalp. This is *habit spasm*, the so-called *false chorea.* With it, we can always tell what movement will occur; whereas, in *true chorea*, we can never predict what the next motion may be. Habit spasm is purely imitative. Then there is the chorea which is of purely hysterical origin, and which is quite distinct from *true Sydenham* chorea. In it we get anesthesia of the eye, the pharynx, or the cutaneous surfaces, which never occur in true chorea. Dr. Putnam here mentioned a case which he had seen at the General Hospital. It was that of a young woman, who, to all appearances, was suffering from genuine chorea. On examination, it was discovered that there existed a total anesthesia of the conjunctiva. This patient was put on a treatment suitable for hysteria, as cold douches, wet sack, etc., and made a prompt recovery. Most cases of true chorea are curable. About fifty per cent.

are due to rheumatism, and there is also a class in which there is a marked neuropathic heredity, especially those of alcoholic parentage.

Chorea from fright is, in Dr. Putnam's opinion, not the true malady. In these cases, we always get a history of night-walking, night-terrors, personal irritability, etc., indicative of hysteria. The pathology of Sydenham chorea is unknown, and the changes described by the older writers certainly cannot exist in it, for they are of a nature prohibiting a recovery in the short time in which true chorea resolves itself. He believes in the relation of chorea with rheumatism, anemia, chlorosis, cerebral irritation, and reflex troubles, as those, according to Dr. Stevens's theory of irregular action of the occular muscles, or eye strains, though these cases are exceedingly rare. Chorea with paralysis, or "limp chorea," is not identical with vulgar chorea. In the latter, we find the muscles normal, while in limp chorea there can always be elicited on examination electrically, the usual degeneration reactions. These cases are very uncommon, and require a totally different treatment, calling at once for the exhibition of strychnine, electricity, etc. The chorea of the old has a bad prognosis, and the same may be said of that form seen in pregnancy. Our treatment must always be fitted to the constitution and condition of the patient, and never by any "rule of thumb."

Dr. Grosvenor spoke of the recent sleeping treatment for chorea, where the patient is made to sleep from twelve to sixteen hours out of every twenty-four. The use of antipyrin has also been highly extolled. Personally, he has used in his practice arsenic and iron.

Dr. Snow spoke of the interest he had always felt in chorea and more particularly in the close and rather mysterious relation between it and rheumatism. In the last fourteen months he had seen nine cases of chorea. Seven of these cases were girls, and two boys,—about the usual proportion between the sexes. In three of these cases the exciting cause appeared to be fright; all of these patients were of the female sex. Six of these cases (sixty-six per cent.) presented a distinct history of rheumatism, three having suffered from severe attacks of the acute articular form. Cardiac lesions were found in five cases, four having systolic murmurs at the apex, and one an aortic direct murmur. One boy suffered from hypertrophy and dilatation, and general dropsy from cardiac weakness. Another, a girl of eleven, had had two attacks of chorea. During the second, an attack of acute articular rheumatism occurred, which was complicated by pericarditis. Chorea was a disease in which relapses were exceedingly common, four of his patients (girls) having a history of more than one attack. Two cases had had two attacks, one four attacks, and another seven. Dr. Snow spoke of the occurrence of rheumatic nodules in chorea, a somewhat unfrequent phase of the rheumatic diathesis, in this country. Alluding to the endocarditis of chorea, he said that all available evidence went to prove that the endocarditis of acute articular rheumatism and that of chorea were identical in respect to pathological changes; that the beaded aspect of the mitral valve in the endocarditis of chorea was merely the primary stage of an endocarditis, and that precisely similar changes were found in rheumatic endocarditis. The vegetations found in valvular inflammations consisted of two elements. First, cell proliferation in the substance of the valve, causing a series of bead-like elevations; and second, a deposition of fibrin on the uneven surface of the valve. Dr. Snow then spoke of the use of arsenic hypodermically, as employed in Vienna, from which he had seen good results.

Dr. ABBOTT was much interested in Stevens's researches on the combination of occular muscular insufficiency, and refractive errors. He believed that not enough attention has been given to Ranney's statement, that eighty per cent. of choreic patients have some occular trouble. The existence of this occular trouble leaves, to say the least, the system in a more susceptible condition. If a case of chorea does not yield readily to treatment, we ought to have the eyes examined.

Dr. F. W. HINKEL said that there are cases of chorea, or choreiform movements, which can be clearly ascribed to nasal or pharyngeal difficulties, as polypi or deflected septa, and are purely reflex in nature. He mentioned a case in which every time the existing nasal hypertrophy was touched certain choreiform movements ensued, but upon removal of the over-growth, all reflex trouble disappeared. Before cocaine was discovered he had often noticed, when operating upon the nose or throat, that some patients exhibited characteristic rolling up of the eyes, and twitching and beating of the feet. Jacobi has observed peculiar muscular twitchings in children, in connection with the existence of naso-pharyngeal adenoid tissue, and which are cured by the removal of the new growths.

Dr. FALK—One the earliest symptoms of chorea is twitching of the eyelids and the alæ of the nose, and which may be so slight as to be easily overlooked. The pupils may be dilated and insensible to light.

The essayists for the January 7th meeting announced are: Dr. Chas. G. Stockton, subject, Some Phases of Glycosuria; and Dr. H. R. Hopkins, subject, Local Treatment of Some of the Local Affections of the Lungs.

